# ‘It feels like my metabolism has shut down’. Negotiating interactional roles and epistemic positions in a primary care consultation

**DOI:** 10.1111/hex.13666

**Published:** 2022-11-16

**Authors:** Olaug S. Lian, Sarah Nettleton, Huw Grange, Christopher Dowrick

**Affiliations:** ^1^ Department of Community Medicine, Faculty of Health Sciences UiT—The Arctic University of Norway Tromsø Norway; ^2^ Department of Sociology University of York York UK; ^3^ Department of Primary Care and Mental Health University of Liverpool Liverpool UK

**Keywords:** epistemic position, epistemic stance, general practice, narrative analysis, patient experience, shared decision‐making

## Abstract

**Introduction:**

Our aim is to explore the ways in which a patient and a general practitioner (GP) negotiate knowledge claims stemming from different epistemic domains while dealing with a mismatch between experiential and biomedical knowledge during the clinical consultation. We interpret their interaction in relation to the sociocultural context in which their negotiation is embedded and identify factors facilitating their successful negotiation (a medical error is avoided).

**Methods:**

Based on a narrative analysis of a verbatim transcript of a complete naturally occurring primary care consultation, we explore the moment‐to‐moment unfolding of talk between the patient and the GP (two women).

**Findings:**

The patient experiences symptoms of what she interprets as a thyroid condition, and indirectly asks for medication. She presents her case by drawing on experiential knowledge (‘it feels like my metabolism has shut down’) and biomedical knowledge (while suggesting a diagnosis and a diagnostic test). The GP informs her that her thyroid blood tests are normal and uses biomedical knowledge to explain why she turns down the patient's request. This stages a potential conflict between the patient's embodied experiential knowledge and the doctor's biomedical knowledge. However, during their encounter, the patient and the GP manage to co‐construct the patient's illness story and make shared decisions about further actions.

**Conclusion:**

The transition from potential conflict to consensus is a result of the mutual efforts of two parties: a patient who persistently claims experiential as well as biomedical knowledge while at the same time deferring to the GP's professional knowledge, and a GP who maintains her epistemic authority while also acknowledging the patient's experiential and biomedical knowledge.

**Patient and Public Contribution:**

Our empirical data are sourced from a data archive and patients were not involved in the design or conduct of the study, but our study is based on a naturally occurring clinical consultation with a patient.

## 1 INTRODUCTION

What furnishes a person's status as a knower? Personal experience for one. There are events, activities and sensations ‘to which the experiencer has primary, sole and definitive epistemic access’.^1^ When we communicate these experiences to others, it is a testimony based on first‐hand knowledge that testifies to the truth of the matter. This means that when patients communicate their illness experiences to doctors in a clinical setting, doctors gain access to ‘testimonially based knowledge’.^2^ Together with knowledge derived from systematic research and clinical experience, patients' testimonial experiential knowledge constitutes a key component in clinical practice.

In the wake of the increasing emphasis on patient‐centred care and shared decision‐making (SDM), it has become increasingly important for doctors to be attentive to patients' experience‐based knowledge. The SDM model, which is founded on a collaborative doctor‐patient relationship and a two‐way exchange of knowledge, means providing patients with decision‐making influence.^3^ To give patients meaningful decision‐making influence, doctors need to be attentive to patients' knowledge and normative stances, and supply sufficient information for patients to be able to make decisions about their healthcare.^4^ Acknowledging patients' expertise, whether experiential or biomedical, is a key prerequisite.

SDM is constrained by the different *institutional* roles and knowledge positions that patients and doctors occupy in clinical consultation.^5^ The medical encounter brings together two ‘territories of knowledge’^1^: the patient's embodied experience and the doctor's biomedical knowledge and technical expertise. The former is subordinated to the latter. It means that while interacting in institutional settings, they do so within a context of epistemic asymmetry.^6^, ^7^, ^8^, ^9^ For each epistemic domain, actors occupy a position on a gradient from knowing to not‐knowing, which they implicitly mark by pointing to ‘presupposed access to knowledge or the rights to knowledge’.^10^ Their institutional epistemic *positions* must be distinguished from their epistemic *stances*, which concerns ‘the moment‐by‐moment expression of these relationships, as managed through the design of turns at talk’.^11^ Positions are fixed, stances are not. People often align their epistemic stance to their epistemic position, but such congruence is not inevitable.^11^ The epistemic primacy of biomedical over experiential knowledge in the medical system severely constrains patients' ability to exercise choice,^12^ and ‘patients' testimonies are often dismissed as irrelevant, confused, too emotional, unhelpful, or time‐consuming’.^6^

Our aim in this study is to explore the ways in which a patient and a GP negotiate knowledge claims stemming from different epistemic domains while dealing with a mismatch between experiential and biomedical knowledge through a case study of one complete naturally occurring clinical consultation. Our analysis involves capturing the ways in which the two parties mark their epistemic positions and stances, and interpreting their knowledge claims in relation to the sociocultural context in which their interaction is embedded. After narratively exploring the moment‐to‐moment unfolding of the consultation, from beginning to end, we reflect on how their interaction might have contributed to solving the potential conflict it entails.

Reducing the epistemic divide between patients and healthcare providers is widely advocated, but SDM is difficult to achieve.^13^ Previous research drawing on naturally occurring consultations points to various ways in which both patients and health professionals are invested in maintaining differences in epistemic authority.^14^ Doctors may limit patients' epistemic access to medical knowledge, for example by providing only interpretations of test results rather than the results themselves,^15^, ^16^ or disguise power by generating *perceptions* of choice.^17^ Patients have been found to deny and downgrade their own knowledge during medical encounters, for example, through the use of epistemic disclaimers like ‘I don't know’,^10^, ^18^ nonconstraining expressions of caution and uncertainty like ‘I was wondering’ and ‘I'm not sure’,^9^, ^19^ or attributions to third parties like ‘my husband thought it could be…’.^9^, ^20^ Particularly in the final decision‐making phases of consultations, patients typically defer to doctors' expertise regardless of their own level of understanding.^18^, ^21^, ^22^, ^23^ Patients who display knowledge in ways that disrupt or resist epistemic asymmetry may be treated as problematic.^24^

Contrary to previous research, we are not focusing on the negative (i.e., epistemic asymmetry) but on the positive, in the sense that we explore how constructive negotiations of knowledge claims across epistemic domains might be successfully achieved in a clinical consultation. Because the onus of achieving SDM is usually placed on healthcare professionals,^4^ patients' role is easily overlooked, and their engagement remains underinvestigated.^25^ We, therefore, emphasize the interactional aspect, and the role of the patient in the decision‐making process.

## 2 METHODS

This is a case study based on a verbatim transcription of a complete naturally occurring primary care consultation, sourced from the *One in a Million: Primary Care Consultations Archive* (Table 1). We chose the case‐study design because of its potential for generating detailed knowledge about complex processes as they occur in their natural setting.^28^

**Table 1:** One in a Million: Primary Care Consultations Archive

Data archive (*n* = 300)	A prospective observational study containing an initial data set archived at the data repository of the University of Bristol, UK. The data set includes 327 film‐ or audio‐recorded and verbatim transcribed naturally occurring GP consultations collected between 2014 and 2015 in 12 National Health Service (NHS) practices in and around the City of Bristol. A total of 300 patients gave informed written consent for their data to be accessed and reused by other researchers, subject to specific ethical approval. The data set also includes patient records; longitudinal patient pre‐ and postconsultation survey data; sociodemographic data of patients and GPs and GP practice data. The *One in a Million* study was funded by the National Institute for Health Research (NIHR) School for Primary Care Research (208) and the South West GP Trust, and received ethics approval from South West—Central Bristol Research Ethics Committee (ref.: 14/SW/0112).^26^, ^27^
Our sample (*n* = 212)	All consultations classified as endocrine/metabolic, neurological, musculoskeletal, psychological, digestive, cardiovascular and general. *Patients*: 135 women and 77 men aged 18–96 (average = 51 years). *GPs*: 13 women and 10 men aged 32–62 (average = 46 years), divided between 12 different practices, who conducted 7–14 consultations each. *Consultations*: 101 consultations were performed with what patients defined as their ‘usual’ GP, 122 were conducted by women GPs and 86 were woman‐to‐woman consultations. All 212 consultations were systematically coded in NVivo (version 12.4) based on a codebook with data‐grounded themes (master‐themes and subthemes) and semantic codes, which means we stayed close to the language of participants and coded what was overtly and explicitly expressed. The codebook, which was generated after coding 25 randomly chosen transcripts (by the first author), was developed collaboratively by the research team, and the final coding was done by the first author and a researcher. We have previously published studies based on larger samples of the data set.^15^, ^18^, ^19^

Abbreviation: GP, general practitioner.

### 2.1 Data material

To identify a consultation where the patient was actively engaged in decision‐making processes, we identified all patients who proposed both interpretations of their condition *and* treatment options (Table 2). After reading all 16 identified consultations, we chose to proceed with a case where the two parties negotiated epistemic positions throughout the whole consultation (Table 3), and therefore was most likely to maximize ‘what we can learn’.^30^ Our patient is averagely engaged in terms of *describing* symptoms and action taken to manage her illness, but above averagely engaged when it comes to making *suggestions* about diagnosis and treatment (Table 2). This is consistent with the case study methodology, where it is common to study an ‘unusual’ case^31^ ‘because of its uniqueness’.^28^ We used the patient record and the patient's responses in the pre‐ and postconsultation surveys as supporting data.

**Table 2:** Patient utterances: Our case compared to the complete data set (selected variables)

Patients	Number of utterances^a^			
	Our case (*n* = 1)	Average (*n* = 212)	Median (*n* = 212)	Range (*n* = 212)
(1) Describe symptoms	14	14	13	0–54
(2) Describe actions taken to manage their illness	9	9	7	0–49
(3) Suggest interpretations of their condition	1	0.3	0	0–10^b^
(4) Suggest treatment initiatives	4	0.6	0	0–5^c^
Total	28	24.5	21	3–99^d^

^a^ Utterances, or speech acts, implying a speaker temporary ‘taking the floor’, are housed in turns at talk, coupled into an alternation of takers.^29^

^b^ Scores identified in 45 (21%) of the 212 consultations.

^c^ Scores identified in 76 (36%) of the 212 consultations.

^d^ In 16 of the 212 consultations, we identified scores on both numbers 3 and 4.

**Table 3:** The consultation

Duration	17 min
Contact reason	Endocrine/metabolic
Patient	Woman in her early 50s, who is ‘Unable to work due to illness’. Education: ‘O‐levels/CSEs/GCSEs or equivalent’ (lowest level).
Patient survey information	The patient answered ‘Strongly agree’ on the questions ‘I know this doctor very well’ and ‘This doctor knows me as a person’.
GP	Woman in her mid‐40s. She is a salaried GP who has worked in her current practice for nearly 9 years. The patient classified her as her ‘usual’ GP.

Abbreviation: GP, general practitioner.

### 2.2 Data analysis

Through this case study, we aim to capture the complexity of a single case and relate its particularities to the institutional setting in which the interaction unfolds. Our first obligation is to understand the presented case. However, because the case represents a social practice in a social institution in which culture is enacted, the case might teach us something about the institution itself. Our study has both an intrinsic and an instrumental approach.^30^

During our analysis, we treated the consultation as a narrative^32^ and explored the complete transcript in relation to *what* was uttered (content), *how* it was uttered (form) and *by whom* (speaker). Our empirical data consist of a dialogue, where meanings emerge through reciprocal exchange. Every utterance is ‘either a statement establishing the next speaker's words as a reply, or a reply to what the prior speaker has just established’.^33^ To preserve context and meaning, while also capturing the ongoing dynamics of the interactional flow, we only worked with dialogue sequences. Our focus on the interactional dynamics is in keeping with Riessman's^32^ performative narrative analysis. By quoting long extracts, analysing components in light of the whole, and attending to sequentiality, we respect the integrity of the narrative.

## 3 FINDINGS

In our selected case, a woman who has experienced a wide range of symptoms for the last 4–5 years meets her ‘usual’ general practitioner (GP). During the consultation, they discuss the patient's symptoms, diagnostic alternatives, causal explanations and options for medical examinations and treatments. Here, we present all key dialogue sequences from these negotiations, in chronological order (Table 4). Each dialogue extract is introduced by a quote from the patient. In the final part, we quote from the GPs entry in the patient record.

**Table 4:** Key content of dialogue extracts

1	The beginning: ‘Yes, I'm fine’
2	The patient moves their conversation to menopause‐related hormone replacement treatment (HRT): ‘The hot flushes are driving me nuts’
3	The patient elaborates on her symptoms: ‘I had a look at the Thyroid UK website thing again’
4	The patient indirectly suggests starting a low dose of thyroxine: ‘A friend of mine did say, “Ask if you can be put on a low dose of thyroxine”’
5	The patient indicates that she is searching for an explanation of her symptoms: ‘I still don't know what the hell is going on’
6	The end: ‘It feels like my metabolism has shut down’
7	Information from patient record 10 days after the visit: ‘Lab results!’

### 3.1 ‘Yes, I'm fine’

The GP starts the consultation with a very common introductory question:

GP: How are you doing?

P: Yes.

GP: Alright? […]

P: Yes, I'm fine. First of all was the result for the [jobbie] yes, which I kept forgetting to phone in about. I'm still old‐fashioned and expect people to phone me back.

GP: Sorry, yes.

P: No, it's not you, I'm saying when they get the results in, if that is what they used to do.

GP: Yes.

P: About thirty years ago I think they did that still. (Laughs)

GP: So that was normal, it was 2.7.

P: Right.

After first refraining from answering the ‘How are you doing?’, the patient adds a brief ‘I'm fine’, before requesting information about a blood‐test result. Though she seems keen to get down to business, she side‐tracks for a moment by indirectly complaining about not being phoned up and informed about these results (which she downplays by labelling herself as old‐fashioned to think like that). Through her clear agenda‐setting at the start, she takes control of the conversation. However, she also intimates lack of biomedical and systems knowledge through the colloquial placeholder ‘jobbie’ (i.e., ‘thing’), and ‘forgetting to phone in’ for the test‐result. At this stage, neither GP nor the patient mentions which test(s) was taken, but later on we learn that it was thyroid‐stimulating hormone (TSH). Too high or too low TSH‐levels indicate that the thyroid is not working correctly. The GP informs the patient that the test result was ‘normal’ and adds the exact figure (2.7), with no further explanation about reference‐ranges for ‘normal’ (which is 0.4 to 4.2 micro‐units per litre).

### 3.2 ‘The hot flushes are driving me nuts’

The patient then abruptly moves their conversation to menopause‐related hormone replacement treatment (HRT):

P: Yes, the thyroid results. Oh yes, I think I need to go back onto the HRT, but I didn't want to do the Premarin. When I ticked the Premarin box, he wouldn't let me have them, which I kind of understood, because it's been about two or three months since I have taken any, and the hot flushes are driving me nuts. But I was remembering what you were saying about—and I thought, because this is so bad now.

GP: I mean, with HRT it is weighing up pros and cons, isn't it? The reality is officially now you're five years you should be taking it, it is five years after the average of the menopause. […] For you, of course, there are many benefits with the osteoporosis [component] side of things. So, I think that's a good idea to go back on it.

P: Yes.

While drawing on her own experiences (‘hot flushes are driving me nuts’) as well as the GP's knowledge and authority (‘I was remembering what you were saying’), the patient proposes resuming HRT. She formulates her request indirectly and modifies it through a subjectifying clause (‘I think I need’). After reminding her that there are pros and cons of this treatment, the GP complements the patient's indirect proposal: ‘I think that's a good idea’ (aligning with the patient's ‘I think’).

### 3.3 ‘I had a look at the Thyroid UK website thing again’

The patient then continues to describe her symptoms, before moving their conversation back to the thyroid issue:

P: I had a friend from [name of American city] come over that I haven't see for eleven years in August. She is my actress friend. For two days of that—she is only here for a week and for two days of it I just got this killer migraine because I'd done a little bit of walking with her. Oh my God, and she is a yoga instructor, so‐

GP: It's frustrating, isn't it?

P: She was trying to help me and stuff, but I can't lift my legs or do anything because of that muscle weakness thing.

GP: Yes.

P: I had a look at the Thyroid UK website thing again because it keeps coming up, and I know that that is coming up normal. So, I thought, ‘Right’, and I saw that they had—I think it's like 45 different symptoms, so I thought, ‘I'm going to write down the ones I've got’, so I did. I've got 32 of them so I thought I'd give them to you so that you've always got them. All of those are still standing.

GP: Yes.

Through the detailed mini‐narrative about the visit from her actress and yoga‐instructor friend, the patient conveys the impact of her symptoms: she spent two of these days with a ‘killer migraine’ just because she had done ‘a little bit’ of walking with her. The GP responds empathically by acknowledging how frustrating that must have been. Then, the patient downplays her own knowledge position by using placeholders (‘thing’ twice) to refer to muscle weakness and a website (Thyroid UK is a charity). Although she signals not knowing official technical terms, she appears to know very well what she is talking about. By collecting online information, she has learned that thyroid conditions might be associated with about ‘45 different symptoms’, of which she experiences 32. She has written down these 32 symptoms so that her GP can ‘always’ refer to them. She confirms she knows that her test ‘is coming up normal’, but a possible thyroid condition is still something she would like to consider because her symptoms tell her something else, which she seems to rely on more. By mentioning the ‘normal’ test results, she pre‐empts a potential objection.

### 3.4 ‘A friend of mine did say, “Ask if you can be put on a low dose of thyroxine”’

The patient then moves their conversation to the issue about further actions:

P: The back thing is just getting worse and worse and worse. A friend of mine did say, ‘Ask if you can be put on a low dose of thyroxine just to see if it does make any difference’. I was wondering if that was going to be at all possible, even though I know that is coming back normal.

GP: Yes, I think that's a difficult one actually.

P: Yes, I know.

Here, the patient proposes to be ‘put on’ a low dose of thyroxine, even though her blood‐tests are normal. She begins indirectly, attributing the suggestion to a friend and using the downtoner ‘just’. Through this ‘displaced authorship’,^20^ she bypasses a direct me‐to‐you challenge of the GPs role by displacing the responsibility of her requests to a third‐party. After quoting her friend, she reformulates the suggestion in her own words, but still expresses it tentatively (‘I was wondering if’). When the GP effectively declines the patient's request, the patient acknowledges the difficulties of her proposal (‘Yes, I know’), but she is not giving up yet.

### 3.5 ‘I still don't know what the hell is going on’

The patient continues her line of argument by giving more details about her symptoms, and reflecting on why they occur:

P: Because I'm getting really weird pains now in this area here and here.

GP: Okay.

P: I don't know if that is a problem of an internal thing, or if that is just the pain radiating out even more because it's getting even worse. So, I'm still not—I still don't know what the hell is going on […]

GP: I guess the other thing is that a lot of these symptoms are also associated with lack of oestrogen. So, like, joint stiffness, the muscles.

P: So, all of these have been going on for what? The last four or five years.

GP: You take oestrogen, I know.

P: But I was taking oestrogen.

GP: I know, yes.

P: I was taking it so that's why I know that a lot of these can be—what's the word I'm looking for? Attributed to other illnesses and God knows what else, but it's when you read it.

GP: Shall we just check it again?

P: What?

GP: Your T4.

P: Oh, right, T4. Is that what I had done?

GP: No, TSH.

P: So, I looked at this one that you can do—I know there are the normal ones that you always do.

GP: Then there is one extra. So T4 and TSH we always do. T3 is an extra. We can try and request a T3. Sometimes—I can successfully now request it, the lab don't process it, but if I phone them up, usually they will then go through. Why don't we try that?

P: Can we?

After describing the pain she is experiencing, and stating baldly that she does not know what is causing it (‘what the hell is going on?’), the GP links it to a lack of oestrogen, albeit tentatively (‘I guess’). The patient objects to the GP attributing her symptoms to lack of oestrogen and reminds her that she has already tried it (‘But I was taking oestrogen’; the ‘but’ probably links to the GPs statement about ‘associated with lack of oestrogen’). Based on her experiences of the oestrogen treatment, she claims to know that these symptoms might be related to other illnesses (‘that's why I *know*’). The GP picks up on this and suggests taking further thyroid tests, through a collaborative doctor‐patient ‘we’ (‘Why don't we try that?’). This appears to be what the patient wants, most of all. When the GP mentions which tests she wants to do (T4 and TSH), the patient takes a more biomedical stance and says she knows that these tests are ‘the normal ones that you always do’, but she also knows about an additional test (‘this one’) that the GP ‘can do’. The GP, who apparently interprets this as a proposal to include other thyroid tests as well, confirms that ‘there is one extra’ they can do, and adds it to the requisition.

In this vital part of the consultation, the patient manages to plead her case effectively, although she repeatedly expresses that she lacks the proper expertise regarding explanations (‘what the hell is going on’ and ‘God knows’), terminology (‘what's the word I am looking for?’) and blood‐tests (‘Is that what I had done?’). However, what she knows and what she claims to know might be two different things. When the patient mentions the test ‘that you can do’, she leaves the GP with one of two options: either offer the patient the extra test or explain why she would not. The GP responds by suggesting that they (again, via the collaborative doctor‐patient ‘we’) request additional thyroid tests, which the patient agrees to (although it was effectively her suggestion).

### 3.6 ‘It feels like my metabolism has shut down’

The GP then moves to explaining why she would not offer the patient the medication she indirectly asked for:

GP: The issue is if we give you thyroxine, it then can have a knock‐on effect and put you into heart failure if you are taking thyroxine when you don't need it.

P: I see, right. I can understand that.

GP: That is the issue, really, because it slightly increases your output so potentially can cause that.

P: So how does it stop the muscle fatigue? How does it help with that? […]

GP: I mean, it's just that your body slows down, so when you become low in thyroxine you come really slow and heavy, you gain weight. […] So as soon as that is too low, the basal metabolic rate goes down, so all your cellular processes are just slowing down.

P: Sure, because that is how I feel.

GP: So, I think that is the theory as to how it causes the fatigue, in the same way as if you take too much, you become very hyper.

P: Yes, yes. Absolutely.

GP: So, I mean, that's the worry, I don't want to give you thyroxine.

P: No, I understand, I understand that.

GP: Unless there is a definite need for it. I mean, but let's check again and see all those three.

P: Yes, of course. I'm also aware that we have only got a short time, but I also did look up—because five years ago when all this started off as well, I had just spent that two years with no sleep. I mean, serious, serious sleep deprivation for two years. So, I did look up online several different sites to see if severe sleep deprivation can trigger hypothyroidism. It says it can do that because it fucks about with your—sorry, with your metabolism. I know that is what has shut down on me. It feels like my metabolism has shut down.

GP: Let's do that, just put on here, ‘Query thyroid disease. T4, TSH and T3 please’.

Throughout the GP's explanations, the patient repeatedly aligns with the GP's stance (‘I can understand that’ and ‘Yes, yes. Absolutely’) and asks for further information (‘How does it help with that?’). While responding to the GP's explanations about ‘metabolic’ and ‘cellular’ processes, the patient claims knowledge by referring to personal experiences (‘that is how I feel’ and ‘it feels like my metabolism has shut down’), and—again—online sources (‘several different sites’). The patient then introduces the medical term ‘hypothyroidism’, which is a diagnosis (meaning the thyroid fails to produce enough thyroid hormone) that the GP has not mentioned, before immediately switching to more informal language (‘it fucks about with your—sorry, with your metabolism’). While drawing on the online information, she presents a possible explanation for why she might have developed this disease (‘severe sleep deprivation can trigger hypothyroidism’). After first concluding that she ‘know[s]’ that her metabolism has ‘shut down’, she quickly reformulates from knowledge to feelings: ‘It feels like my metabolism has shut down’.

### 3.7 ‘Lab results!’

The consultation ends with a prescription for a hormone patch to ease menopause symptoms, and a referral for four different blood‐tests (T4, TSH and T3 plus an antibody test). Ten days later, the entry in the patient record reads: ‘Lab results! Thyroid autoantibodies [AB], agreed to T4 as AB raised—but very unusual with normal TSH […] make nonurgent appt with GP’, which means that the patient has an autoimmune thyroid condition in need of thyroxine treatment.

## 4 DISCUSSION

In the presented consultation, the patient describes experiences of symptoms that she interprets as a thyroid condition, but her interpretation is not supported by biomedical findings (her blood‐tests are normal). The mismatch between the patient's embodied experiential knowledge and the doctor's biomedical knowledge stages a potential conflict. The absence of a diagnosis means that the patient is not receiving appropriate medical treatment, which she now seeks professional help to get. For the GP, the mismatch complicates her dual obligation: to acknowledge patients' experiential knowledge, and to make decisions based on the most up‐to‐date reliable scientific evidence. It is easy to imagine a negative outcome here (thyroid condition going undiagnosed for years) because the experiential is still so often subordinated to the biomedical.

Instead, a medical error is avoided. So, what works in this case?

### 4.1 Balancing experiential and biomedical knowledge

The answer lies in the interaction between the patient and the GP. The contribution from the patient is essential. She states the purpose of her visit in their very first exchange, and she continues to control the agenda‐setting until the very end by providing information, asking questions, presenting her views and proposing actions. While building her case, she draws on a range knowledge sources. While describing how she experiences her symptoms and their implications, she treats herself as entitled to experiential knowledge. This is how it *is*. When she claims to be entitled to know because of what she experiences, she marks her epistemic stance. Building on her own symptom descriptions, she presents a candidate diagnosis (hypothyroidism), a possible explanation (long‐term sleep deprivation) and a possible course of actions (a supplementary diagnostic test and medical treatments). In addition to her embodied experiences, she draws on several knowledge‐sources: (a) what the GP previously said, (b) what other people have said and (c) various online sources.

By claiming biomedical knowledge, the patient shows she has done her research. However, she adapts her knowledge‐claims to the recipient^34^ by continually talking as if she speaks to a person with epistemic primacy: (1) she downplays her own biomedical knowledge, and marks it as the GP's domain (waiting for the GP to fill in correct terms and correcting herself from ‘know’ to ‘feel’); (2) she modifies her proposals with lexical downtoners^35^ (‘just’); (3) she marks her proposals as tentative or subjective by embedding them within other clauses (‘I think’) and (4) she asks for permission (‘I was wondering if that was going to be at all possible’). By doing so, she aligns her epistemic stance to her epistemic position, and acts within an implicit framework in which the decisive decision‐making power is placed with the GP. In downplaying her own knowledge, she talks as though she is not allowed epistemic access to biomedical understandings of illness, and not allowed to share decision‐making with the GP. This is a remnant of the pre‐SDM era that indicates a cultural lag, where new institutional ideals about patient‐centred care are not yet internalized by the patient. By not challenging the GP's authoritative medical position, she avoids being confrontational.^10^, ^18^, ^20^ This might have been significant for the outcome: doctors do not consider it helpful, and may become annoyed, if patients insist on their preferences and doubt their doctors' recommendations.^36^

Although the patient is careful not to challenge the GP's expertise by aligning her epistemic stance to her subordinate epistemic position, her outright claim to biomedical knowledge is of vital importance for the outcome of the consultation. The most forceful of these statements is seen in extract 3.5, when the patient refers to a blood‐test not yet taken that the GP ‘can do’. By making clear that she knows about this test, the GP either has to offer her the test or explain why she would not. Given the patient's experiential testimony about her symptoms and their implications, it would be difficult for the GP not to offer her the test, which eventually leads to a correct diagnosis and appropriate medical treatment.

The GP contributes to the positive result (a medical error is avoided) by allowing the patient to talk and listening attentively to what she says, while also expressing understanding and sympathy. It is potentially difficult to spot the toned down, understated and hedged utterances that the patient makes, but this GP detects them and responds with respect. The importance of understanding that patients are constrained by their institutional position, and detecting and attending to patients' downtoners, is a key lesson of this study. The GP maintains her epistemic authority throughout (e.g., initially rejecting the patient's indirect treatment proposal), but she remains open to the patient's contribution, engages with her proposals despite their indirectness and reconsiders the available evidence based on the patient's knowledge‐claims. When she provides professional opinions based on biomedical knowledge, it is always with an openness to complexity and uncertainty (repeatedly ‘think’ but also ‘guess’; ‘weighing up pros and cons’ and ‘that's a difficult one’). By not closing their debates before the topics are thoroughly discussed, she facilitates patient engagement.

All these communicative aspects are likely to facilitate patient engagement, patient‐centred care and SDM. To further enhance our knowledge about such interactional factors, we need more research on positive interactions in naturally occurring consultations, more studies with ‘naturalistic’ designs (in contrast to experimental and hypothetical), and more case study research.

The collaborative and consensus‐orientated interaction that we see in the presented consultation is of course not only a result of what happened in the consultation room that particular day. Previous research indicates that when patients meet their ‘usual’ GPs, as our patient does, there may be more opportunities for them to resist epistemic asymmetry.^14^ Their apparently open, honest and respectful dialogue indicates mutual trust, which clearly contributes to the positive outcome of their interaction: By combining experiential and biomedical knowledge, the patient and the GP manage to co‐construct the patient's illness‐story and make mutual decisions about further actions.

### 4.2 Strengths and limitations

Our empirical data give us a unique opportunity to explore doctor–patient interaction in situ. By doing an in‐depth analysis of a single case, we are able to explore in detail the moment‐to‐moment unfolding of a complete consultation as it occurs in its natural setting. Working with observation‐data, however, prevents us from asking participants to elaborate their utterances, and our only information about what happens outside the consultation room comes from the patient record and her responses to the pre‐ and postconsultation surveys. Possible biases in the data relate to recruitment of GPs, who self‐selected to take part in the study,^27^ and participants might have been influenced by their awareness of being filmed.

## 5 CONCLUSION

The presented consultation is indicative of how a patient and a GP who face a mismatch between experiential and biomedical knowledge manage to use a mix of knowledge‐sources to co‐construct the patient's illness story and share decision‐making responsibility. Although both parties largely align their epistemic stance to their epistemic position (one speaking the language of ‘knowing’, the other of ‘feeling’), they manage to merge them: the patient finds her symptoms in the GP's description of hypothyroidism (‘Sure, because that is how I feel’), and the GP takes the patient's experiential and biomedical knowledge seriously enough to consider that the test results received so far may not be telling the whole story (‘let's check again’). The transition from potential conflict to consensus is a result of the mutual efforts of two parties: a patient who persistently claims experiential as well as biomedical knowledge without dismissing the expertise and authority of the GP, and a GP who acknowledges not only the patient's experience‐based knowledge but also her biomedical knowledge.

## CONFLICT OF INTEREST

The authors declare no conflict of interest.

## ETHICS STATEMENT

The One in a million study received ethics approval from South West—Central Bristol Research Ethics Committee (ref.: 14/SW/0112). All participants (patients and GPs) gave informed written consent for their data to be accessed and reused by other researchers, subject to specific ethical approval. Our study received ethics approvals from the National Health Service (Research Ethics Committee reference 18/WM/0008; Integrated Research Application System project ID 232578), and Bristol Data Repository clearance from the Data Access Committee. All data were anonymized upon receipt, and there was no contact with study participants.

## Data Availability

Data sharing is not applicable to this article as no new data were created or analyzed in this study. The data used in this study are sourced from the One in a Million: Primary care consultations archive (https://www.bristol.ac.uk/primaryhealthcare/researchthemes/one-in-a-million/one-in-a-million/). Restrictions apply to the availability of these data, which were used under license for this study.
